# Quantitative levels of aripiprazole parent drug and metabolites in urine

**DOI:** 10.1007/s00213-014-3781-1

**Published:** 2014-10-28

**Authors:** Joseph McEvoy, Robert A. Millet, Kenneth Dretchen, Ayodele A. Morris, Michael J. Corwin, Peter Buckley

**Affiliations:** 1 Medical College of Georgia, 3411 Kamel Circle, Augusta, GA 30909 USA; 2 Carolina Behavioral Care, 4102 Ben Franklin Blvd, Durham, NC 27704 USA; 3 Department of Pharmacology and Physiology, Georgetown University Medical Center, 3900 Reservoir Road NW, Washington, DC 20057 USA; 4 Ameritox, Ltd., 486 Gallimore Dairy Road, Greensboro, NC 27409 USA; 5 Care-Safe, LLC, 24 Crescent St, Waltham, MA 02453 USA; 6 Medical College of Georgia, Georgia Regents University, 1120 15th Street, AA 1002, Augusta, GA 30912 USA

**Keywords:** Aripiprazole, Dehydroaripiprazole, OPC3373, Urine drug testing

## Abstract

**Objective:**

The aim of this study is to assess urine levels of aripiprazole and metabolites among patients receiving steady-state dosing of aripiprazole.

**Methods:**

One hundred fifty adults, judged compliant with a stable aripiprazole regimen, had observed dosing for 5 consecutive days. Urine specimens, obtained on days 1, 4, and 5, were analyzed for pH, creatinine, specific gravity, and for aripiprazole, OPC3373, and dehydroaripiprazole. Linear regression was used to assess the association between unadjusted urine levels of each drug/metabolite and dose taken, and linear stepwise multiple regression was performed to identify variables that added to the explanation of the variance.

**Results:**

OPC3373 was found in 97 % of urine samples, whereas unchanged aripiprazole and dehydroaripiprazole were found in only 58 and 39 % of samples, respectively. Variance in urine metabolite levels accounted for by medication dose was relatively low for each individual drug/metabolite, *r* ^2^ only 0.13 to 0.23. However, when OPC3373 was adjusted for age, weight, sex, and urine creatinine values, the *r* ^2^ improved to 0.63, and further improved to 0.70, when height, urine specific gravity, and the presence of dehydroaripiprazole were added in a stepwise multiple regression model.

**Conclusions:**

Unadjusted urine levels of aripiprazole and metabolites are not strongly related to aripiprazole dosing, however, accounting for key variables yields a strong relationship between measurable urine parameters and dose taken. By defining the expected range of adjusted urine levels for each dose, the potential exists for a clinical test to identify partially nonadherent individuals who would not have been identified by conventional “present vs. absent” urine drug testing.

Adherence to a medication regimen plays an important role in maximizing outcomes for individuals with schizophrenia, depression, and other mental health disorders (Valenstein et al. 2001; Velligan et al. 2003; Weiden and Glazer 1997). Medication plan adherence is poor across a wide variety of physical and psychiatric conditions (Dolder et al. 2002; Grymonpre et al. 1998; Haynes 1979; Velligan et al. 2003), and it is especially poor in patients with schizophrenia (Velligan et al. 2003; Weiden and Glazer 1997; Lindenmayer et al. 2009). It has been estimated that half of patients with schizoaffective disorder and schizophrenia take less than 70 % of their prescribed dose (Cramer and Rosenheck 1998). Poor adherence (including partial adherence) has been found to be an independent predictor of treatment discontinuation (Perkins et al. 2008), as well as relapse, re-hospitalization, long-term functional outcome, and suicide in patients with schizophrenia (Llorca 2008). Although new generations of drugs, such as aripiprazole, are becoming increasingly available with improved side effect profiles, levels of adherence remain alarmingly low (Grymonpre et al. 1998; Velligan et al. 2003).

Over the last few decades, the literature has described many possible explanations for the causes of poor adherence and presented potential approaches to improving compliance (Fenton et al. 1997; Oehl et al. 2000; Zygmunt et al. 2002). Unfortunately, there has been remarkably little agreement regarding an objective definition of adherence, or how to measure it. Current techniques are either not accurate enough, prone to error, or very difficult to perform in the clinical setting.

The most common method used to assess adherence in the mental health population has been patient self-report (Velligan et al. 2006, 2010). In a study by Velligan et al. (2007) comparing patient self-report or physician assessment of compliance with more objective measures, it was shown that neither patients nor physicians were able to characterize adherence accurately. The objective measures used in the study were pharmacy fill rates of patients’ prescriptions, pill counts, and electronic monitoring. Although more accurate, these measures would probably not be easy to perform in clinical practice for a variety of reasons (Byerly et al. 2007; Farmer 1999). It has been a matter of debate whether serum therapeutic drug monitoring (TDM), a more invasive method, should be implemented in clinical practice (Hiemke et al. 2011). Serum TDM is a powerful tool for determination of adherence for some antipsychotic medications. Despite increasingly sophisticated methods to measure adherence, alternative objective tools could improve ascertainment (Sajatovic et al. 2010). Once nonadherence is identified, proven strategies to improve adherence can be employed (Velligan and Sajatovic 2013).

As with other therapies, urine drug testing (UDT) for antipsychotic medication therapy is commercially available to test for the presence or absence of the drug. The limitations of traditional qualitative UDT have been noted (Nafziger and Bertino 2009). Detection of partial nonadherence, which is likely more common than taking no doses, is especially challenging since a positive urine test may occur even with substantial, clinically relevant partial nonadherence. Quantitative UDT has been in use for some time primarily for pain medication management, where lower than expected urine drug levels is a factor in evaluating not only partial nonadherence but also diversion or hoarding of medication, and higher than expected levels can be used to assess overdosing which can lead to hospitalization and death.

The goal of the current study was to assess the relationship between aripiprazole dose taken and quantitative urine levels of aripiprazole and its metabolites, among patients receiving steady-state dosing of aripiprazole. Importantly, we also wished to determine if the strength of this relationship would be improved if we accounted for variables that have the potential to impact on urine levels of aripiprazole and its metabolites. It is hoped that these data may lead to a quantitative UDT that can help clinicians understand patient compliance, at a level beyond what can be achieved by simple present vs. absent testing, and become an efficient, clinically and cost-effective tool for physicians who treat these patients.

## Methods

### Study population

This prospective study was conducted from 03/11/2013 to 12/17/2013 at Carolina Behavioral Care in Durham, Hillsborough and Pinehurst NC and at Georgia Regents University in Augusta, GA. IRB approval was obtained for all study sites.

The study population consisted of 150 adult outpatients who, in the clinical judgment of the study staff, were compliant with a stable once daily aripiprazole regimen for 2 weeks prior to enrollment. Recruitment was stratified with a goal of enrolling between 30 and 60 subjects in each of the following three ranges of aripiprazole dosing: (a) 2–5 mg, (b) 10–15 mg, and (c) 20–30 mg. Exclusion criteria were as follows: known to be pregnant or breastfeeding, known significant hepatic or renal impairment, a significant medical condition that would interfere with study participation, or ingestion of certain concomitant medications and foods over the past 2 weeks that interfere with metabolism of aripiprazole.

After providing informed consent, subjects underwent an abbreviated physical exam and provided a medical and medication history. On each of five consecutive days, all subjects were required to come to the clinic, where study staff observed as each subject took her/his prescribed dose of aripiprazole. On study days 1, 4, and 5, the subjects provided a pre-dose urine specimen. On study day 5, subjects were evaluated for disease severity using the Clinical Global Impression (CGI) (Guy 1976). All urine samples were collected just prior to drug administration, near the nadir of serum/urine drug levels (median time between prior dose and urine sample 24.1 h, extremes 17.9 and 30.7 h; and median time between urine sample and next dose was 5 min, extremes 1 and 109 min. The average time of dosing was 11:30 a.m. (extremes 7:00 a.m. and 1:00 p.m.).

### Laboratory methods

Upon receipt at Ameritox, Ltd. (Greensboro, NC), urine specimens underwent immediate specimen validity tests for pH, creatinine, and specific gravity. This was followed by confirmatory analysis for aripiprazole metabolites, OPC3373 and dehydroaripiprazole, and parent drug, aripiprazole. The authentic specimens were prepared by fivefold dilution with deionized water acidified with formic acid, and methanolic internal standard solution. Urine samples were analyzed on a Waters Acquity UPLC TQ MS (Waters, Milford, MA) using a Waters Acquity UPLC® CSH™ Phenyl-Hexyl, LC analytical column (2.1 mm × 50 mm, 1.7 μm). The internal standard solution contained aripiprazole D8 and clozapine D4. Subject samples were not hydrolyzed prior to analysis. The column temperature was held at 50 °C and the injection volume was 5 μL. The mobile phase consisted of 2 mM ammonium acetate with 0.1 % formic acid (solvent A) and methanol (solvent B). Analytes were separated by gradient elution in a 3.6 min total cycle time. Mass spectral data was acquired in positive electrospray ionization mode with two selected transition ions for all analytes and internal standards. The source temperature was 150 °C and desolvation temperature was 600 °C. The desolvation gas was nitrogen and the collision gas was argon with flow rates of 1200 L/h and 0.20 mL/min, respectively. Cone gas flow was 100 L/h. Multipoint calibration curves were prepared in normal human urine in the established linear range for each analyte and at the same dilution as specimens. The limits of quantitation/detection (LOQ/D) were 5 ng/mL for aripiprazole and dehydroaripiprazole and 25 ng/mL for OPC3373. Upper limits of linearity and carryover were 5000 ng/mL for all compounds. Inter- and intra-assay precision did not exceed 11.0 % coefficient of variation, and accuracy was within 16.6 % of target concentrations for all compounds.

### Statistical methods

The proportion of all urine samples for which each metabolite was detectable was tabulated to verify data reported previously (Dretchen et al. 2013) that the OPC3373 metabolite was the only metabolite that was consistently detectable in subjects taking aripiprazole. Based on this observation, the urine OPC3373 value was the primary factor used in equation development.

Based on first pharmacokinetic principles, an equation was developed to calculate an adjusted OPC3373 level in an attempt to account for individual physiological and anatomic differences between the study subjects. The adjuster equation used the raw urine OPC3373 metabolite concentration, age, weight, sex, and urine creatinine values.

When calculating the adjusted OPC3373 level, if a subject had an OPC3373 concentration below the LOQ (i.e., 25 ng/mL), a value of 12.5 ng/mL (i.e., halfway between zero and 25) was used for the adjustment, since it is not possible to calculate a natural log (Ln) for zero.

Correlation coefficients were calculated for the adjusted OPC3373 levels for days 1 vs. 4, 1 vs. 5, and 4 vs. 5 to assess the stability of values across study days and to determine the suitability of pooling results from urine samples obtained on the different study days. Note that the first 20 subjects enrolled in this study also had serum aripiprazole levels obtained on days 1 and 5, and based on these data, we have previously reported that stability in serum levels from day 1 to 5 was consistent with the subjects being adherent to their prescribed dosing at study entry and at steady state during the period of observed dosing.

When pooling results, multiple results from the same subject were weighted such that there was an equal contribution to the analysis from each subject (i.e., if a subject contributed two samples, each received a weight of 0.5, whereas if a subject contributed only one sample, the sample received a weight of 1.0). Linear regression methods were used to assess the association between unadjusted urine levels of each drug/metabolite and observed dose taken.

A linear stepwise multiple regression was performed using the natural log of the dose as the dependent variable and a number of candidate independent variables in an attempt to identify those that significantly added to the explanation of the variance. The candidate variables included adjusted urine OPC3373 levels, presence vs. absence of urine aripiprazole and dehydroaripiprazole, urine creatinine, urine specific gravity, urine pH, age, sex, race, smoking category (never, past, or current) height, weight, and time between urine test void and prior void. Variables were added to the model as long as the addition of that variable added to the *r* ^2^ at *p* < 0.15. In an attempt to create a final parsimonious model, only variables that improved the *r* ^2^ by >0.015 were included in the final model.

## Results

### Study population

The characteristics of the 150 subjects enrolled in the study are shown in Table 1. Approximately two-thirds of the subjects were female, and the mean age was 44.6 years. The most common psychiatric diagnoses were major depressive disorder (41.3 %) and bipolar disorder (37.3 %). Only 16.0 % of subjects had schizophrenia. Consistent with these diagnoses, the mean illness severity on the CGI was 2.6.

**Table 1:** Characteristics of subjects

	All subjects
Characteristic	*N* = 150
Male, *n* (%)	61 (34.0)
Age (years), mean (SD)	44.7 (12.0)
Race, *n* (%)	
Caucasian	90 (60.0)
Black	51 (34.0)
Other/Unknown	9 (6.0)
Hispanic, *n* (%)	3 (2.0)
Marital status, *n* (%)	
Single	56 (37.3)
Married	35 (23.3)
Separated/divorced	47 (31.3)
Living with partner	3 (2.0)
Widowed	9 (6.0)
Education, *n* (%)	
< HS	33 (22.0)
HS graduate	40 (26.7)
Some college	55 (36.7)
College graduate	22 (14.7)
Employment status, *n* (%)	
Unemployed	39 (26.0)
Part-time employment	21 (14.0)
Full-time employment	11 (7.3)
Student	4 (2.7)
Retired	14 (9.3)
Disabled	61 (40.7)
Cigarette smoking, *n* (%)	
Current	84 (56.0)
Past	23 (15.3)
Never	43 (28.7)
Current psychiatric diagnosis^a^, *n* (%)	
Schizophrenia	24 (16.0)
Bipolar disorder	56 (37.3)
Major depressive disorder	63 (41.3)
Other	52 (34.7)
Clinical global impression, mean (SD)	2.6 (1.1)
Body mass index, mean (SD)	31.8 (8.4)

^a^Subjects may have had more than one psychiatric diagnosis

**Fig. 1:**
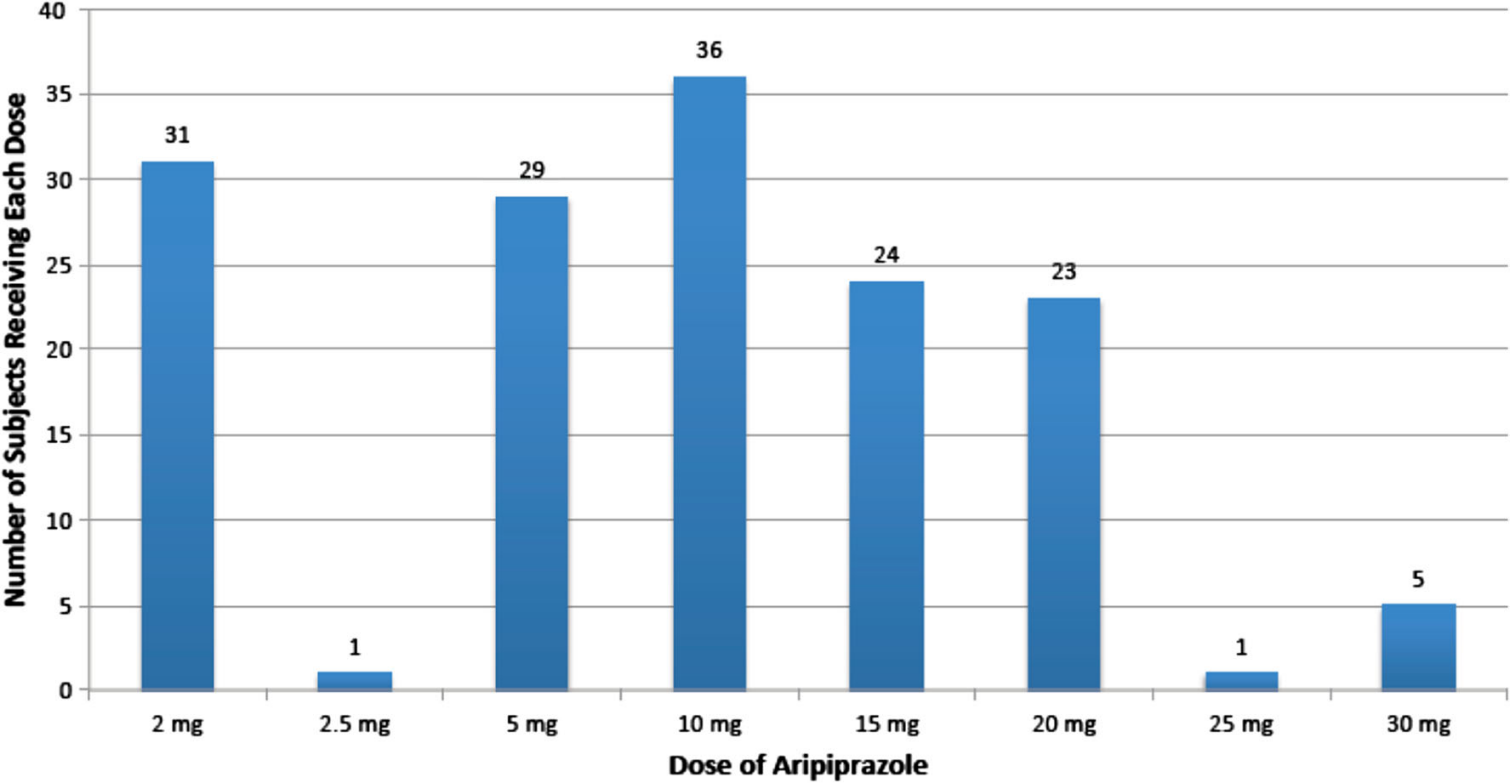
Number of subjects receiving each aripiprazole dose (*n* = 150)

The distribution of aripiprazole doses taken by subjects is shown in Fig. 1. The stratified enrollment strategy resulted in 61 subjects in the low-dose stratum (2–5 mg), 60 in the middle-dose stratum (10–15 mg), and 29 in the higher dose stratum (20–30 mg).

#### Analyses of each metabolite in urine samples

Six urine samples from two subjects (one taking a dose of 2 mg, and one taking a dose of 5 mg) were excluded from analyses because the subjects were found to be ineligible following enrollment as they were taking excluded medications. Two additional subjects did not provide all three urine samples; one provided a urine sample only on day 1, the other only on days 1 and 4, leaving a total of 441 urine samples available from 148 subjects (148 day 1 samples, 147 day 4 samples, and 146 day 5 samples).

**Fig. 2:**
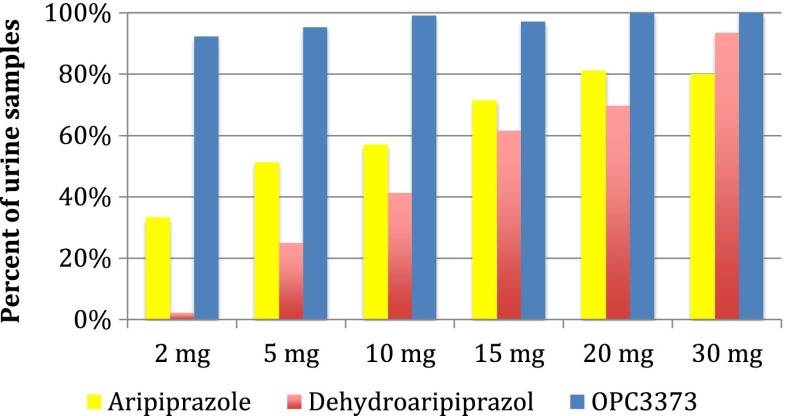
Percent of samples greater than the limits of detection for each metabolite by dose (note that data for 2.5 and 25 mg doses were omitted from the figure due to the small number of samples at those doses)

OPC3373 was the metabolite most consistently present in urine samples (Fig. 2). OPC3373 was found in (97 %) urine samples overall, ranging from 92 % of samples from individuals taking 2 mg to 100 % of samples from those taking 20 mg or greater. Whereas, unchanged aripiprazole and dehydroaripiprazole were found in only 58 and 39 % of urine samples, respectively, with relatively low rates of detection even at higher doses.

**Fig. 3:**
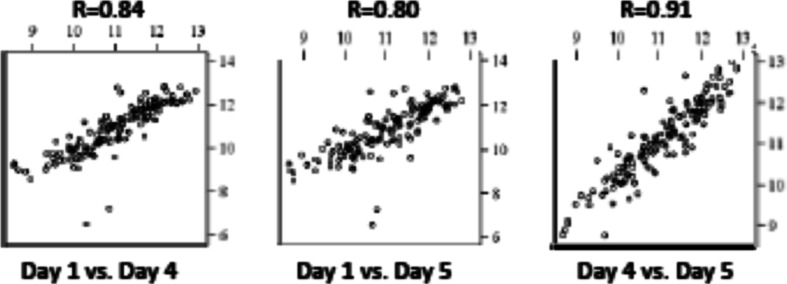
Correlation between adjusted urine OPC3373 levels across dosing days

There was high correlation between adjusted urine OPC3373 levels in urine samples obtained on days 1, 4, and 5. The strongest correlation was between days 4 and 5 (*R* = 0.91), with the correlations between days 1 and 4 and 1 and 5, being only slightly lower (0.84 and 0.80), respectively (Fig. 3). These results suggested that the goal of enrolling adherent individuals on stable dosing was achieved, with a very small number of lower adherent individuals, likely accounting for the slightly lower correlation between the day 1 levels vs. day 5, compared to the day 4 levels vs. day 5. Based on the high correlation between days 4 and 5 adjusted urine OPC3373 levels, days 4 and 5 urines were pooled, so that there were 293 samples used in subsequent analyses.

#### Relationship between unadjusted urine metabolites and aripiprazole dose

**Fig. 4:**
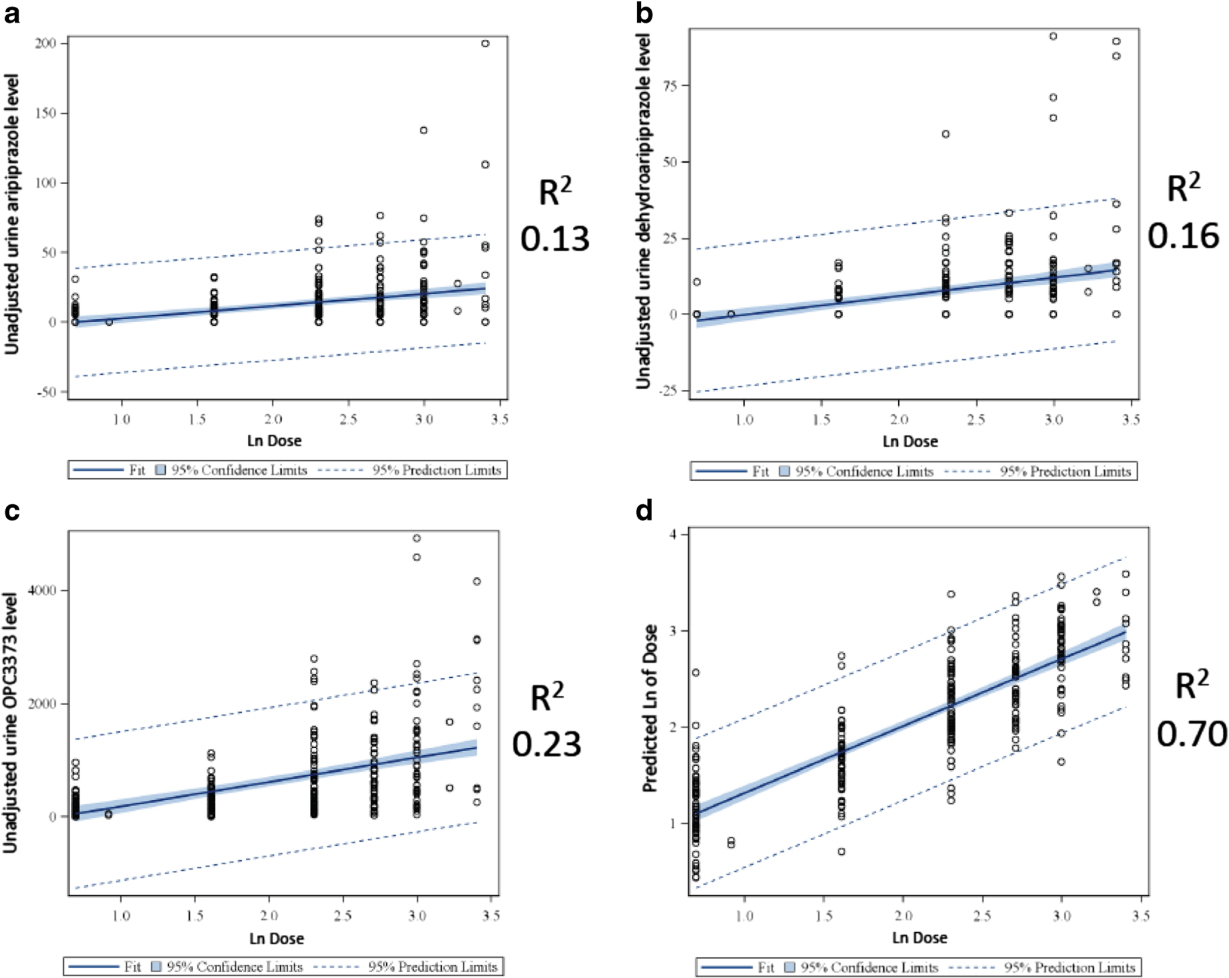
**a** Unadjusted urine aripiprazole vs. ln dose. **b** Unadjusted urine dehydroaripiprazole vs. ln dose. **c** Unadjusted urine OPC3373 vs. ln dose. **d** Predicted ln dose from multiple regression equation vs. ln dose

The relationship between each individual drug/metabolite and aripiprazole dose taken is shown in Fig. 4a–c. The variance in urine metabolite levels that is accounted for by medication dose, as measured by the *r* ^2^, is relatively low for each individual drug/metabolite, ranging from only 0.13 for aripiprazole to 0.23 for OPC3373.

#### Relationship between adjusted urine OPC3373 plus other variables and aripiprazole dose

The results of the stepwise multiple regression analyses are shown in Table 2. Adjusted OPC3373 was the most important single variable with an *r* ^2^ of 0.63. Five other variables met the criteria of *p* < 0.15 for inclusion in the stepwise regression, with height adding an additional *r* ^2^ of 0.03 to the model, and urine specific gravity and the presence of dehydroaripiprazole in the urine, each adding an additional 0.02 to the model. Although, race and time between urine test void and last prior void, both met our initial criteria for inclusion in the model, each added only 0.01 or less to the *r* ^2^ value, and in the interest of having a reasonably parsimonious model, we elected to use only the variables that contributed at least 0.02 to the *r* ^2^ in the final model which is illustrated in Fig. 4d, with a final model *r* ^2^ of 0.70.

**Table 2:** Results of stepwise multiple linear regression model: partial *r* ^2^ and model *r* ^2^ for natural log of aripiprazole dose

Independent variable	Partial *r* ^2^	Model *r* ^2^	*P* value
Adjusted urine OPC3373	0.63	0.63	<0.0001
Height (inches)	0.03	0.66	<0.0001
Urine specific gravity	0.02	0.68	0.0001
Dehydroaripiprazole present in urine	0.02	0.70	<0.0001
Black race	0.01	0.71	0.0015
Time between urine test void and prior void	0.003	0.71	0.11

## Discussion

This study has shown that while unadjusted urine levels of aripiprazole and two of its metabolites are not strongly related to aripiprazole dosing, when multiple drug/metabolites are taken into account and when one accounts for physiologic variables that may impact on the urine metabolite levels, there is a strong relationship between measurable urine parameters and dose taken. This offers the potential for clinicians to identify individuals who appear adherent based on standard present vs. absent testing, but who have urine results that are lower than expected for their prescribed dose.

We have previously reported, based on the first 20 subjects enrolled in this study (Dretchen et al. 2013), that at doses of 2–5 mg, OPC3373 was the most consistently identified urine drug/metabolite of aripiprazole, being found in 93 % of samples, compared to 50 and 8 % for urine aripiprazole and dehydroaripiprazole, respectively. These findings have been confirmed and expanded with the full sample of 150 subjects to show that overall OPC3373 was detectable in 97 % of samples (ranging from 92 % at 2 mg to 100 % at doses >15 mg), whereas urine aripiprazole and dehydroaripiprazole were detectable less frequently, even at high doses (percent detected ranging from 33 % at 2 mg to 80 % at 30 mg for aripiprazole, and 2 % at 2 mg to 93 % at 30 mg for dehydroaripiprazole). It should be noted, however, that we enrolled only a small number of individuals, with severe disease (e.g., schizophrenia), who were taking aripiprazole doses of >20 mg, and therefore have a limited ability to draw conclusions about this higher dose range.

In humans, aripiprazole is primarily converted in the liver to two major metabolites. It undergoes dehydrogenation to form dehydroaripiprazole, which is pharmacologically active. It is also converted through dealkylation to form the inactive compound OPC3373. These pathways involve both CYP2D6 and CYP3A4 enzymatic pathways. Less than 1 % of aripiprazole is excreted unchanged in the urine. Blood levels of aripiprazole have been shown to be increased in individuals with hepatic impairment (Mallikaarjun et al. 2008). As expected, the blood levels of the dehydroaripiprazole derivative are reduced in liver toxicity (Mallikaarjun et al. 2008). Results of the same study revealed that the blood levels of aripiprazole were increased during renal impairment (Mallikaarjun et al. 2008). The mean elimination half-lives for aripiprazole and its other active metabolites are 75 and approximately 94 h, respectively. Steady-state serum levels are achieved in 14 days of dosing (Otsuka America Pharmaceutical, Inc. 2014). This study enrolled subjects that were judged to be adherent to a stable dosing regimen and utilized a 5-day observed dosing period to improve confidence that we knew the aripiprazole dose was actually taken by the subject. Although we cannot be certain that subjects were indeed always adherent prior to enrollment or that they did not take additional aripiprazole at home during the observed dosing days, the high correlation between the days 1, 4, and 5 samples suggests that subjects were indeed on stable doses and, especially by day 4 of observed dosing, were at steady state. In the first 20 subjects of this study, who had days 1 and 5 serum aripiprazole levels, steady-state dosing was also supported by stability of serum aripiprazole levels over the 5-day observed dosing period (Dretchen et al. 2013).

Our findings regarding unadjusted levels of aripiprazole in urine are not dissimilar to the findings regarding unadjusted levels of clozapine in blood. Although the utility of measuring clozapine levels in blood is established as a means of TDM, it is known that there is substantial inter- and intraindividual variability because of variability of CYP-450 composition as well as changing daily caffeine intake and smoking; clinicians must incorporate these additional factors into dosing decisions.

The importance of understanding adherence clinically cannot be overstated. Of primary importance is achieving an effective dose of medication to alleviate patients’ mental health symptoms. When adherence with medication is unknown, it is difficult for the physician to know when to adjust the dose or to change to another drug. Failure to detect nonadherence using insensitive methods may lead to delayed identification and offers the risk of relapse (Velligan and Sajatovic 2013).

Monitoring medication adherence by conventional “present vs. absent” urine drug testing is limited to the identification of individuals whose urine drug levels are below the LOD (e.g., 5 ng/mL for aripiprazole and dehydroaripiprazole; 25 ng/mL for OPC3373). Our data demonstrate that such testing using unadjusted aripiprazole and dehydroaripiprazole may often provide false negative results, since even during observed dosing, individuals commonly have urine concentrations for these metabolites that are below the LOD.

OPC3373 is clearly the most appropriate metabolite for assessment of adherence, since it was almost always above the LOD in these subjects who had observed dosing. However, although OPC3373 performed well for assessment of “present vs. absent” testing, with few false negatives, the unadjusted OPC3373 levels did not change very much as the dose of aripiprazole changed. In contrast, with the adjustment algorithm developed in this study, the association between urine levels and dose was much greater, such that it is possible to identify a range of values for which the urine value would be above the LOD (i.e., present), but be clearly below the expected level for a specified prescribed dose. Therefore, by defining the expected range of the adjusted urine levels for each dose, the potential exists for a clinical test that would identify partially nonadherent individuals who would not have been identified by conventional “present vs. absent” urine drug testing. This distinction would likely have substantial clinical utility, as intermittent adherence is an important clinical obstacle to appropriate pharmacologic treatment with antipsychotic medications.
